# The pattern recognition receptors dectin-2, mincle, and FcRγ impact the dynamics of phagocytosis of *Candida*, *Saccharomyces*, *Malassezia*, and *Mucor* species

**DOI:** 10.1371/journal.pone.0220867

**Published:** 2019-08-08

**Authors:** Mohammed Haider, Ivy M. Dambuza, Patawee Asamaphan, Mark Stappers, Delyth Reid, Sho Yamasaki, Gordon D. Brown, Neil A. R. Gow, Lars P. Erwig

**Affiliations:** 1 Medical Research Council Centre for Medical Mycology at the University of Aberdeen, Aberdeen Fungal Group, Institute of Medical Sciences, Foresterhill, Aberdeen, United Kingdom; 2 Division of Molecular Immunology, Medical Institute of Bioregulation, Kyushu University, Fukuoka, Japan; 3 Department of Molecular Immunology, Medical Mycology Research Center, Chiba University, Chiba, Japan; University of Birmingham, UNITED KINGDOM

## Abstract

Phagocytosis is a receptor-mediated process critical to innate immune clearance of pathogens. It proceeds in a regulated sequence of stages: (a) migration of phagocytes towards pathogens, (b) recognition of PAMPs and binding through PRRs, (c) engulfment and internalisation into phagosomes, (d) phagosome maturation, and (e) killing of pathogen or host cells. However, little is known about the role that individual receptors play in these discrete stages in the recognition of fungal cells. In a previous study, we found that dectin-2 deficiency impacted some but not all stages of macrophage-mediated phagocytosis of *Candida glabrata*. Because the C-type lectin receptor dectin-2 critically requires coupling to the FcRγ chain for signalling, we hypothesised that this coupling may be important for regulating phagocytosis of fungal cargo. We therefore examined how deficiency in FcRγ itself or two receptors to which it couples (dectin-2 and mincle) impacts phagocytosis of six fungal organisms representing three different fungal taxa. Our data show that deficiency in these proteins impairs murine bone marrow-derived macrophage migration, engulfment, and phagosome maturation, but not macrophage survival. Therefore, FcRγ engagement with selective C-type lectin receptors (CLRs) critically affects the spatio-temporal dynamics of fungal phagocytosis.

## Introduction

Phagocytosis is a receptor-mediated process in which innate immune phagocytes, such as dendritic cells, neutrophils, and macrophages, engulf and process cargo in membrane-bound vacuolar compartments termed phagosomes [1]. This process is highly dynamic and proceeds in a sequence of stages: (a) migration of phagocytes towards pathogens; (b) recognition of pathogen-associated molecular patterns (PAMPs) and binding by pattern recognition receptors (PRRs); (c) engulfment and internalisation into phagosomes; (d) phagosome maturation; and (e) killing of host or pathogen cells [2]. Pathogen recognition is mediated by receptors of various classes, including immunoglobulin G (IgG) Fc gamma receptors (FcγRs), Toll-like receptors (TLRs), NOD-like receptors (NLRs), and C-type lectin receptors (CLRs). Of these, CLRs in particular have been shown to be critical for antifungal innate immunity [3, 4]. They constitute a superfamily of heterogeneous soluble transmembrane proteins defined by a C-type lectin domain (CTLD) that facilitates binding to major carbohydrate structures found in the cell walls of nearly all medically relevant fungi [5]. The target epitopes are primarily β-glucan and mannose-based structures [3].

With over a thousand members identified so far [4], CLRs have been categorised into various groups based on their structure and signalling capacity. Those that transduce signals upon fungal recognition and binding have been subjects of intense study, and include dectin-1, dectin-2, mannose receptor (MR), and mincle [6]. Signalling through these receptors results in a multitude of outcomes, including phagocytosis, cytokine/chemokine secretion, production of soluble inflammatory lipids [7], and modulation of Th1 and Th17 adaptive immune responses [8]. Transduction of signals by dectin-1 family members is mediated by virtue of intrinsic immunoreceptor tyrosine-based activation motif (ITAM)-like motifs (hemITAMs) in their cytosolic domains, whereas dectin-2 family members (dectin-2, mincle, MCL) couple to the Fc receptor common gamma chain (FcRγ) [9, 10]. Importantly, this coupling is essential for optimal cell surface expression and function, and is also shared by receptors of other classes, such as receptors for IgG (*e*.*g*. FcγRII/III). A shared feature of the dectin-1 and dectin-2 signalling pathways is that they converge on the Syk-PKCδ-CARD9-Bcl10-MALT1 axis to activate NFκB [8] and mediate an array of cellular responses.

Phagocytosis is a crucial and early consequence of CLR signalling. A complex interplay of factors influences the eventual outcome of this process. Ideally, pathogens are contained, processed, and killed, and are therefore prevented from dissemination. However, many pathogens have evolved mechanisms to evade recognition and clearance. For example, *Histoplasma capsulatum*, the causative agent of life-threatening histoplasmosis, blocks V-ATPase-dependent acidification of the phagosome lumen, and therefore impairs phagosome-lysosome fusion and phagolysosome formation [11]. *Aspergillus terreus*, a less frequent cause of invasive bronchopulmonary aspergillosis (IBPA), is remarkable in that it persists in macrophage phagosomes despite low pH [12]. *Candida albicans*, the fourth most frequent cause of nosocomial bloodstream infections [13], activates a number of stress-response pathways (*e*.*g*. Hog1, Cap1, and Hsf1) [14, 15] to counteract the hostile microenvironment of the phagosome, and also extrudes ammonia to neutralise the phagosome and induce hyphal growth [16].

Previously, we have shown that several fungal-specific factors impact on the dynamics of *C*. *albicans* phagocytosis. These include fungal cell viability, cell wall glycosylation status, hyphal length, and spatial orientation [2, 17–19]. However, little is known about how the engagement of particular CLRs affects the spatio-temporal dynamics of fungal phagocytosis. We recently reported that dectin-2 deficiency compromised phagocytic uptake and killing in *ex vivo* models of systemic *C*. *glabrata* infection [20]. Because dectin-2 couples to FcRγ for optimal expression and function, we hypothesised that FcRγ coupling may be crucial for the regulation of phagocytosis dynamics in fungi. We therefore set out to address this hypothesis using not only *Candida* spp. as model cargo, but also fungi from other major fungal taxons: Ascomycota (*C*. *albicans*, *C*. *auris*, *Saccharomyces cerevisiae*), Basidiomycota (*Malassezia dermatis*, *M*. *globosa*), and Mucoromycotina (*Mucor circinelloides*). Using bone marrow-derived macrophages from wild-type mice and mice deficient in the FcRγ adaptor chain, as well as two FcRγ-coupled CLRs (dectin-2 and mincle), our live cell imaging experiments identified a critical role for FcRγ coupling in regulating the dynamics of fungal phagocytosis. Compared with wild-type macrophages, macrophages lacking FcRγ itself, dectin-2 or mincle, were impaired in migration, engulfment, and phagosome maturation, but their viability was largely unaffected. These data therefore highlight a crucial role for FcRγ-coupling in regulating discrete stages of fungal phagocytosis.

## Materials and methods

### Mice

Mice used in this study were in a C57Bl/6 genetic background, and were typically sacrificed at 8 to 12 weeks of age for experimental work. Knockout strains included dectin-2^-/-^ [21], mincle^-/-^ [22], and FcRγ^-/-^ [21]. Mice were bred and maintained in individually ventilated cages at the Medical Research Facility at the University of Aberdeen. All mice were handled in strict accordance to institutional and Home Office guidelines.

For generating bone marrow single cells, femurs and tibia were harvested from mice euthanised using carbon dioxide inhalation followed by cervical dislocation. Animal work was covered by project license number 70/8073 (G. D. Brown). Animal experiments conformed to the animal care and welfare protocols approved by UK Home Office (project license P79B6F297) in compliance with all relevant local ethical regulations.

### Growth and culture of fungal organisms

*Candida albicans* serotype A strain CAI4-CIp10 (NGY152) [23] was grown from -80°C glycerol stocks and plated on YPD plates. YPD medium was prepared by dissolving 10 g yeast extract (Oxoid), 20 g Mycological Peptone (Oxoid), 20 g D-glucose (Fisher Scientific), and 20 g Technical agar (Oxoid) in 1 L distilled H_2_O. Single colonies from YPD plates were cultured in YPD broth and incubated overnight at 30°C and 200 rpm. The following day, fungal cells were washed three times at 3300 *g* and then resuspended in PBS.

Single colonies of *C*. *auris* (CBS 10913T) [24] and *Saccharomyces cerevisiae* (S288c) [25] were obtained from glycerol stocks and cultured similarly to *C*. *albicans* above.

*Mucor circinelloides* (CBS 277.49) [26] spores were obtained from glycerol stocks and grown on solid PDA at room temperature for 7 days. Spores were collected by adding 10 ml PBS and gently scraping into suspension. Harvested spores were filtered through a 40 μm cell strainer, centrifuged at 6000 rpm for 5 min, and washed twice in PBS.

*Malassezia dermatis* (CBS 9169) [27] and *Malassezia globosa* (CBS 7966a) [28] were obtained from glycerol stocks and grown on solid Modified Dixon media. The latter consisted of 36 g malt extract (Oxoid), 10 g Bacteriological Peptone (Oxoid), 20 g Oxoid bile salts (Oxoid), 10 ml Tween-40 (Acros Organics, Geel, Belgium), 2 ml glycerol (Acros Organics), 2.85 ml oleic acid (FisherBrand), and 15 g Technical agar (Oxoid) dissolved in 1 L distilled H_2_O. Cells were grown at 35°C for 7 days. For experiments, colonies were used to inoculate Modified Dixon broth, and were incubated at 30°C and 200 rpm for 1 to 4 days to obtain sufficient growth. Cells were then centrifuged at 3300 *g*, washed three times, and resuspended in PBS. Heat killing of cells was carried out as previously described [20] when required.

### Preparation and culture of murine bone marrow-derived macrophages (BMDM)

BMDM were prepared by flushing the tibia and femurs of 8-12-week-old male or female C57Bl/6 wild-type, dectin-2^-/-^, mincle^-/-^, or FcRγ^-/-^ mice with RPMI 1640 1X + GlutaMax (Thermofisher Scientific, Paisley, UK) supplemented with 10% (v/v) foetal bovine serum (Sigma, Dorset, UK) and 200 U/ml penicillin-streptomycin (Sigma) through a 25–27 G needle. Bone marrow cells were collected in 50 ml Falcon tubes, resuspended, and then filtered through a 70 μM cell strainer. Filtered suspensions were centrifuged at 400 *g* for 5 min, after which they were plated on bacteriological plastic plates (Falcon, PA, USA) in BMDM medium at 37°C and 5% CO_2_. The latter consisted of 1x IMDM (Iscove’s Modified Dulbecco’s Medium) (with L-glutamine and 25 mM HEPES, Thermofisher Scientific), 30% v/v L929 cell-conditioned medium [29], 10% v/v foetal bovine serum (Sigma), 1X minimum essential medium non-essential amino acids (Thermofisher Scientific), and 200 U/ml penicillin-streptomycin (Sigma). BMDM medium was aspirated and replaced with fresh, pre-warmed medium at 3, 6, and 7 days after plating. BMDM were maintained thereafter up to at least 3 weeks by replacing BMDM medium every two to three days.

For use in assays, BMDM medium was aspirated and replaced with PBS containing 4 mg/ml Lidocaine-HCl and 10 mM EDTA (both from Sigma). Cells were incubated at 37°C for 10 min or until cells started detaching from the surface, and then vigorously pipetted and collected in 50 ml Falcon tubes. Cells were quenched with equal volumes of BMDM media, and then centrifuged at 400 *g* for 5 min. Following cell counts, BMDM were seeded appropriately in different plates depending on the experiment and in the absence of L929 cell-conditioned medium.

### Immunostaining of cells with anti-lysosome-associated membrane protein 1 (LAMP-1) antibody

To quantify phagosome maturation, BMDM were prepared as described previously and co-incubated with different fungi at an MOI of 3:1 (fungus: macrophage = 3 X 10^5^ cells/well: 1 X 10^5^ cells/well) for either 45 or 180 min on μ 8-well glass-bottomed slides (Ibidi, Martinsried, Germany). Cells were washed thrice in PBS and fixed in 2% paraformaldehyde for 10 min at 4°C. After three more washes, cells were permeabilised in 0.25% w/v saponin (Sigma) and 1% w/v BSA in PBS, and incubated for 30 min at room temperature. After three washes in PBS, cells were blocked in 10% w/v skimmed milk powder and 0.3% v/v Tween-20 (Fisher Bioreagents, Loughborough, UK) in PBS for 1 h at room temperature. Blocking buffer was aspirated and replaced with blocking buffer containing monoclonal mouse anti-human Alexa-Fluor 488-conjugated anti-LAMP1 IgG1 antibody (Biolegend, London, UK) at 10 μg/ml. Cells were incubated for 1 h at room temperature. After three washes, cells were bathed in PBS and imaged on an UltraVIEW VoX spinning disk confocal microscope (Nikon, Surrey, UK) using an electron multiplying charged coupled device (EMCCD) camera. Imaging data were analysed using Volocity 6.3.1 software (Improvision, PerkinElmer, Coventry, UK). At least 50 phagosomes per condition of treatment were selected randomly for analysis. An unpaired *t*-test was used to analyse data for statistical differences.

### Phagocytosis assays and live cell imaging

BMDM were seeded one day before imaging at 1 X 10^5^/well in 8-well slides. On the next day, assays were performed as described previously [19]. Briefly, BMDM medium was aspirated and replaced with pre-warmed, supplemented CO_2_-independent medium (ThermoFisher Scientific) containing various fungi at an MOI of 3:1 in the presence of 50 nM LysoTracker Red DND-99 (LTR) and 1 μM YOYO-3 Iodide (all from ThermoFisher Scientific). LTR stains all acidified cellular compartments. YOYO-3 is cell impermeable, and only fluoresces when bound to nucleic acids. It is therefore a marker of general cell death. Cells were incubated at 37°C and 5% CO_2_ and imaged for 6 h with an UltraVIEW VoX spinning disk confocal microscope using an EMCCD camera. Imaging data were analysed using Volocity 6.3.1 software. At least three independent experiments were performed for each fungal organism with BMDM from at least three different WT, dectin-2^-/-^, mincle^-/-^, or FcRγ^-/-^ mice. From each movie, at least 100 macrophages were randomly selected and subsequently analysed.

### Analysis of live cell imaging movies

Volocity 6.3.1 software was used to analyse live cell imaging data. The analytical package enabled the tracking of thousands of individual BMDM. Measurements made automatically include distances travelled, track lengths, meandering indices, directionality, and velocity. In this study, mean track velocities and mean displacements of individual BMDM co-cultured with various fungal organisms were calculated and data were represented graphically as tracking diagrams. Manual measurements included: time of initial cell-cell contact; time at which fungal cells were fully engulfed; engulfment time (calculated by subtracting the time point at which initial cell-cell contact occurred from the time point at which fungal cells were fully internalised); phagosome maturation (defined as the time from completion of engulfment till localisation with LTR); and percentage of macrophage death (as depicted by % YOYO-3 positivity).

### Statistical analyses

Data were analysed on GraphPad Prism 5.04 (GraphPad Software, Inc., USA) and expressed as means ± SEM of at least three independent experiments. Statistical significance was assessed by one-way /two-way analysis of variance (ANOVA) or unpaired *t*-tests followed by the Bonferroni multiple comparisons test where appropriate. Data were considered statistically significant where *p* < 0.05. Where appropriate, *n* values were given in figure legends as *n* = A, B, C, D, where *n* was the number of macrophages/phagosomes analysed, A corresponded to WT, B corresponded to dectin-2^-/-^, C corresponded to mincle^-/-^, and D corresponded to FcRγ^-/-^ BMDM.

## Results

### Deficiency in FcRγ, dectin-2, or mincle impairs macrophage migration and displacement

**Fig 1:**
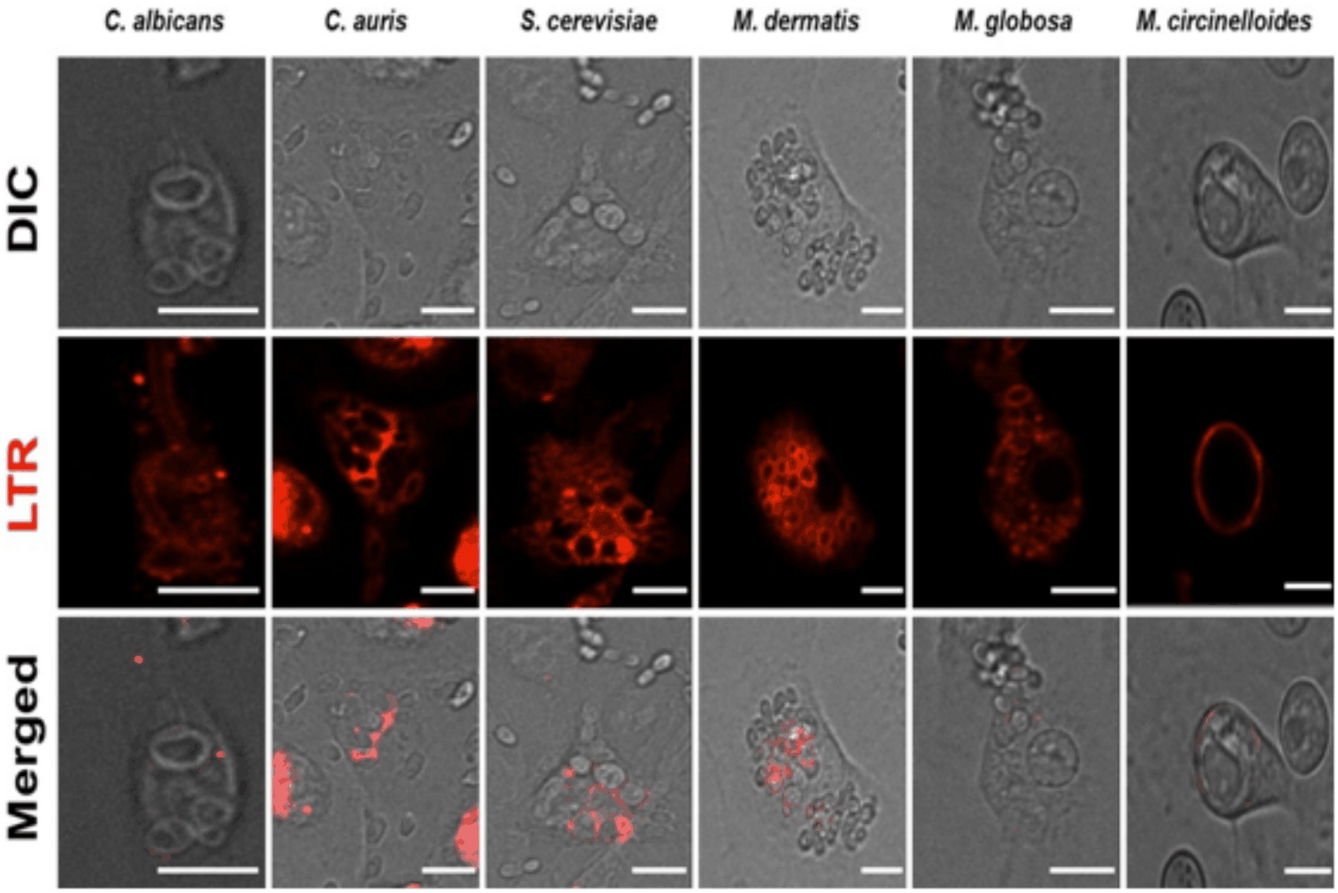
Sample representation of live cell video microscopy. A sample representation of live cell video microscopic images is given. BMDM were routinely co-incubated with six different fungi and imaged for 6 h. Individual panels show BMDM that have phagocytosed different fungal organisms and show phagosome localisation with LTR. Scale bar: 10 μm.

Live-cell video microscopy of macrophages co-incubated with the selected fungi was performed using established protocols [30, 31] (Fig 1). Track measurements were taken at 2 min intervals over the initial 45 min of each video. Tracking diagrams were constructed to follow the movement of thousands of individual macrophages, where each track represents the movement of a single macrophage, symbols indicate its position at 2 min intervals, and arrowheads portray directionality (S1–S6 Figs).

**Fig 2:**
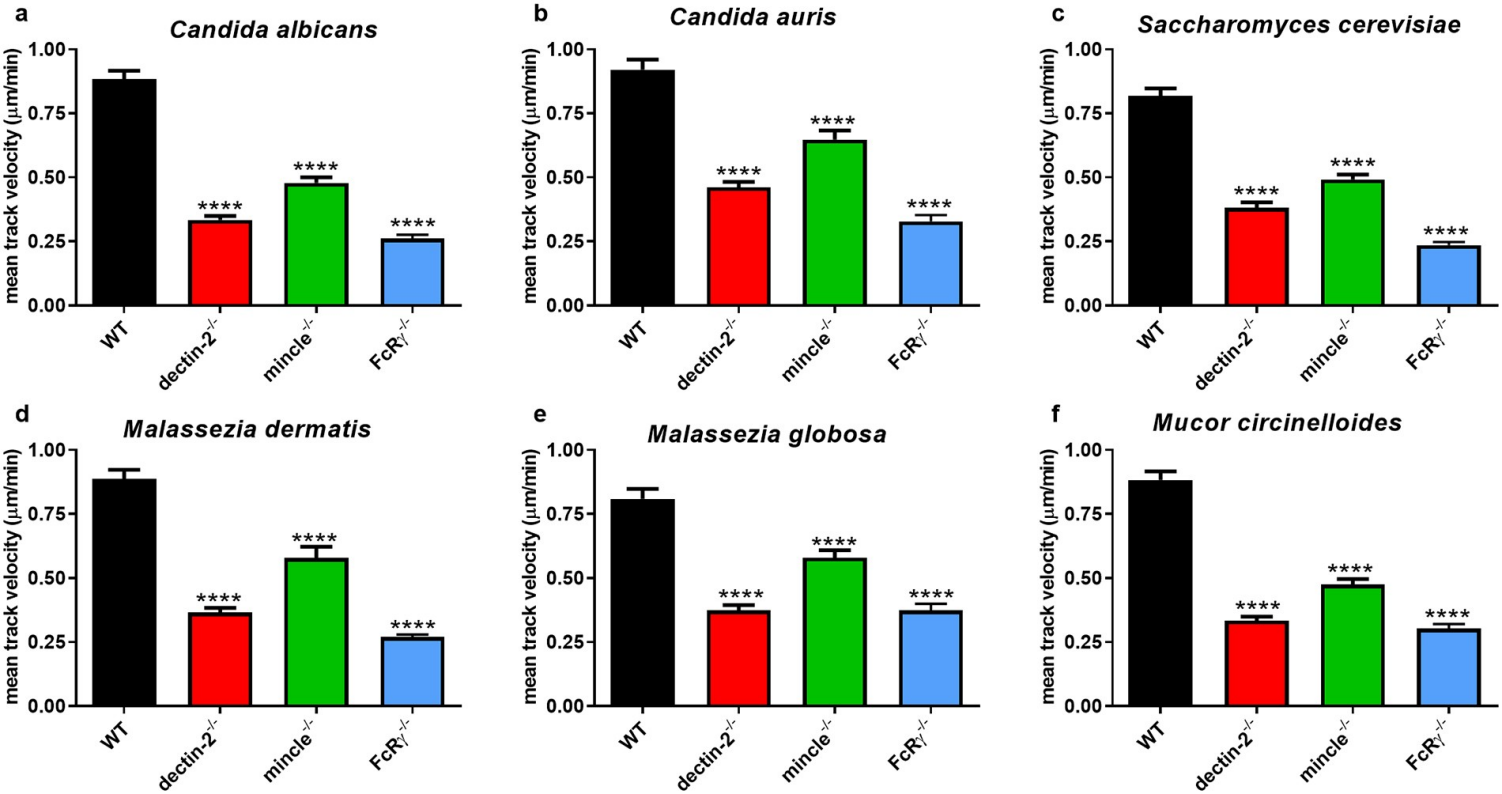
Deficiency in FcRγ or its associated receptors dectin-2 and mincle correlates with impairment of BMDM migration. BMDM from WT, dectin-2^-/-^, mincle^-/-^, or FcRγ^-/-^ mice were inoculated with (a) *C*. *albicans* (*n* = 160, 149, 191, 120), (b) *C*. *auris* (*n* = 162, 166, 205, 119), (c) *S*. *cerevisiae* (*n* = 155, 150, 172, 127), (d) *M*. *dermatis* (*n* = 164, 153, 182, 140), (e) *M*. *globosa* (*n* = 151, 167, 174, 135), or (f) *M*. *circinelloides* (*n* = 160, 149, 165, 244) and imaged for 6 h. Data show the mean track velocity (μm/min) ± SEM of macrophages from three independent experiments. *n* = pooled number of macrophages analysed across three independent experiments per fungal organism. **** *p* < 0.0001 (one-way ANOVA).

Migration is defined as the mean track velocity of macrophages. Deficiency in dectin-2, mincle, or FcRγ correlated with significantly impaired macrophage migration in comparison with WT BMDM, and this was true for all six organisms examined (*p* < 0.0001) (Fig 2).

**Fig 3:**
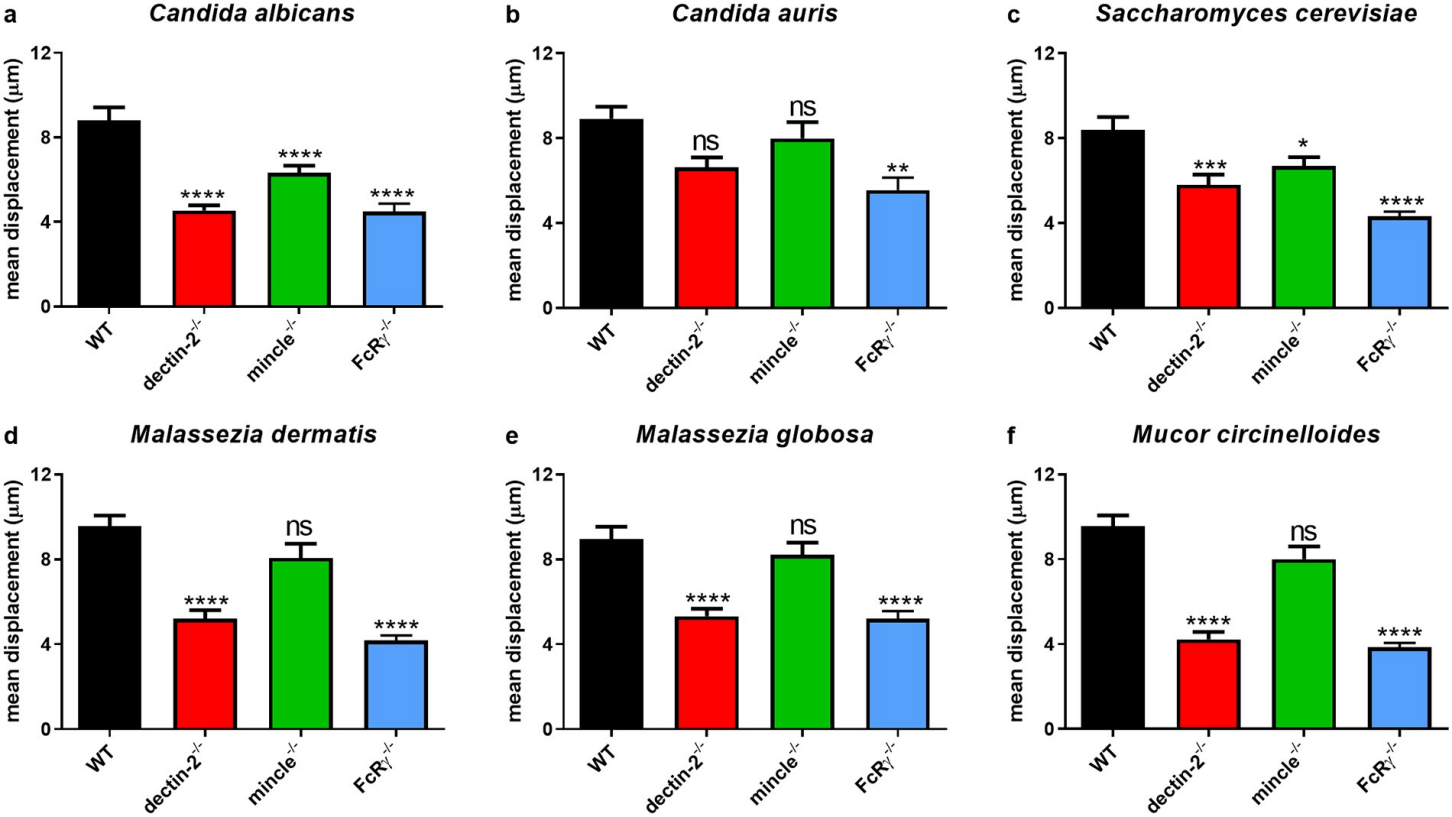
Deficiency in FcRγ or its associated receptors dectin-2 and mincle correlates with impairment of BMDM displacement. BMDM from WT, dectin-2^-/-^, mincle^-/-^, or FcRγ^-/-^ mice were inoculated with (a) *C*. *albicans* (*n* = 149, 154, 186, 120), (b) *C*. *auris* (*n* = 161, 164, 203, 119), (c) *S*. *cerevisiae* (*n* = 155, 147, 171, 127), (d) *M*. *dermatis* (*n* = 160, 147, 181, 140), (e) *M*. *globosa* (*n* = 151, 166, 174,135), or (f) *M*. *circinelloides* (*n* = 160, 145, 164, 244) and imaged for 6 h. Data show the mean displacement (μm) ± SEM of macrophages from three independent experiments. *n* = pooled number of macrophages analysed across three independent experiments per fungal organism. ** *p* < 0.01, *** *p* < 0.001, **** *p* < 0.0001 (one-way ANOVA).

Quantitative analysis also showed significant differences in the mean displacement of BMDM (Fig 3). This is defined as the mean of the distance between an individual macrophage’s physical origin at *t* = 0 and final position for the first 45 min of each video. FcRγ^-/-^ BMDM exhibited significantly impaired displacement in all fungal co-cultures compared with WT BMDM. Displacement of dectin-2^-/-^ BMDM was significantly impaired compared with WT BMDM in all fungal co-cultures except with *C*. *auris* (Fig 3b). Mincle^-/-^ BMDM displacement was only significantly impaired in co-cultures with *C*. *albicans* (Fig 3a) and *S*. *cerevisiae* (Fig 3c). These data suggest that deficiency in FcRγ, dectin-2, or mincle resulted in varying degrees of impairment of macrophage migration and displacement in these fungal co-cultures.

### Deficiency in FcRγ, dectin-2, or mincle delays engulfment of fungal organisms

**Fig 4:**
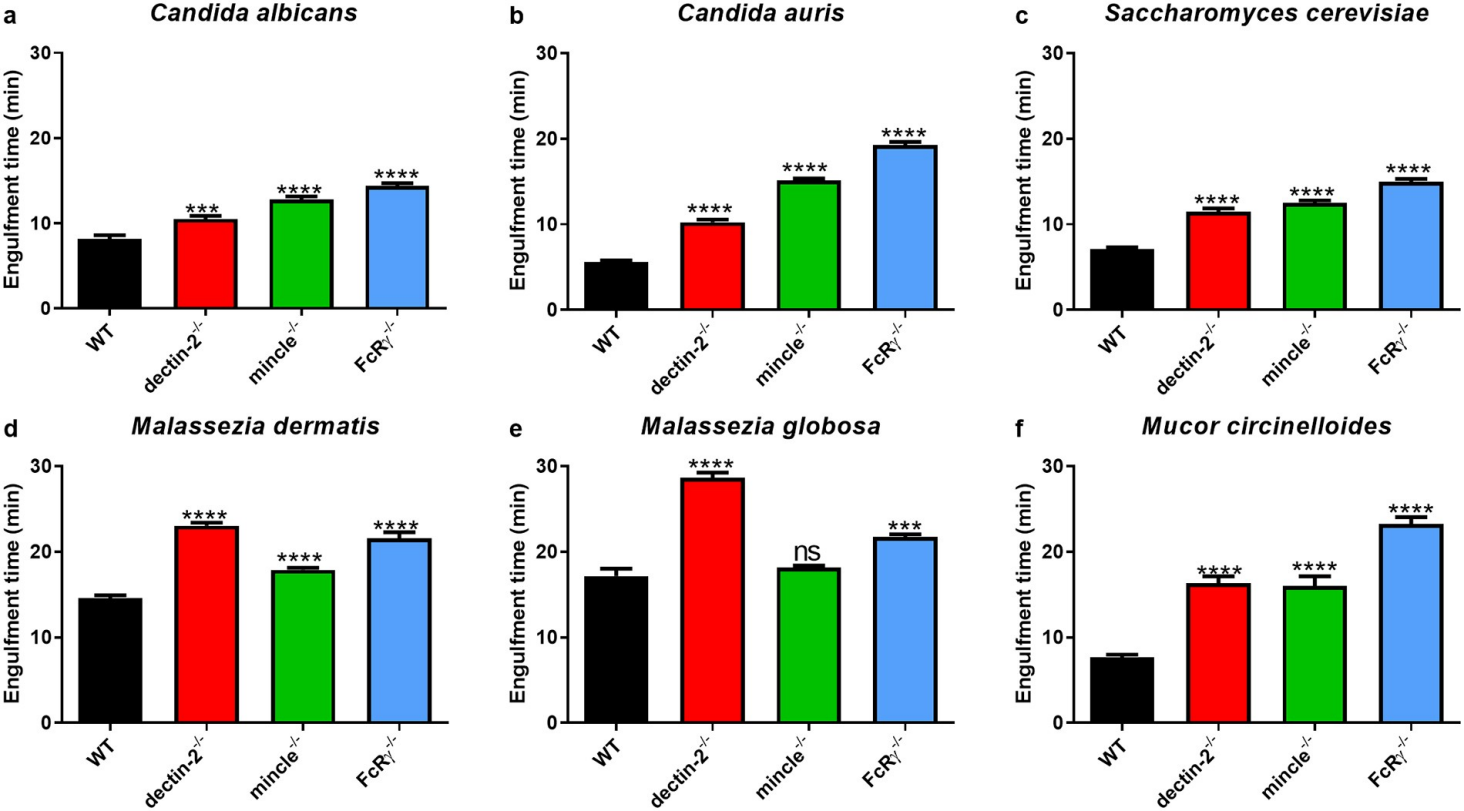
Deficiency in FcRγ or its associated receptors dectin-2 and mincle correlates with delayed engulfment of fungal cells. BMDM from WT, dectin-2^-/-^, mincle^-/-^, or FcRγ^-/-^ mice were inoculated with (a) *C*. *albicans* (*n* = 286, 293, 406, 373), (b) *C*. *auris* (*n* = 346, 358, 1104, 387), (c) *S*. *cerevisiae* (*n* = 280, 230, 325, 283), (d) *M*. *dermatis* (*n* = 798, 886, 1103, 299), (e) *M*. *globosa* (*n* = 717, 914, 927, 444), or (f) *M*. *circinelloides* (*n* = 133, 107, 158, 102) and imaged for 6 h. Data show the mean engulfment time (min) ± SEM of fungal target cells and are from three independent experiments. *n* = pooled number of phagosomes analysed across three independent experiments per fungal organism. At least 50 macrophages were analysed per mouse per fungal organism. ns = not significant, *** *p* < 0.001, **** *p* < 0.0001 (one-way ANOVA).

Having observed an effect for FcRγ-coupled CLR deficiency on migration and displacement, we next examined the effects on the subsequent stage of phagocytosis: engulfment. In this context, engulfment time is defined as the time from initial point of contact between a macrophage and a fungal cell until complete internalisation into a phagosome. Compared with WT BMDM, dectin-2^-/-^, mincle^-/-^, and FcRγ^-/-^ BMDM were significantly impaired in engulfing most of the fungi examined, with the strongest effects seen for mincle^-/-^ and FcRγ^-/-^ BMDM (Fig 4). The only exception to this observation was for mincle^-/-^ BMDM in co-cultures with *M*. *globosa* (Fig 4e), where no differences were observed. Overall, these data suggest that deficiency in FcRγ, dectin-2, and mincle delayed BMDM engulfment of fungal organisms.

### Deficiency in FcRγ, dectin-2, or mincle delays phagosome maturation

**Fig 5:**
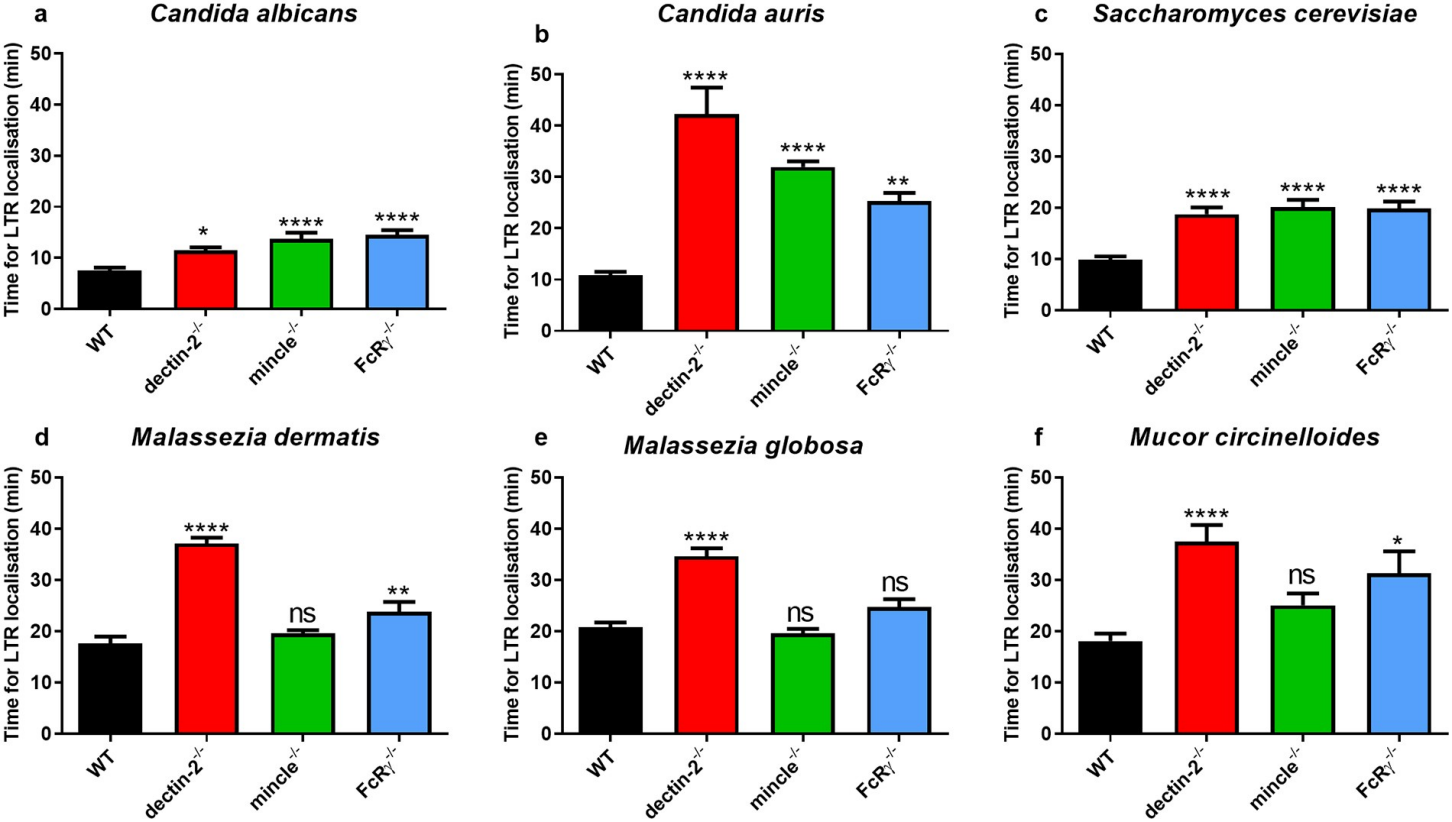
Deficiency in FcRγ or its associated receptors dectin-2 and mincle correlates with delayed phagosomal LTR localisation. BMDM from WT, dectin-2^-/-^, mincle^-/-^, or FcRγ^-/-^ mice were inoculated with (a) *C*. *albicans* (*n* = 264, 267, 349, 298), (b) *C*. *auris* (*n* = 350, 366, 977, 287), (c) *S*. *cerevisiae* (*n* = 276, 201, 284, 231), (d) *M*. *dermatis* (*n* = 618, 760, 991, 264), (e) *M*. *globosa* (*n* = 545, 804, 820, 409), or (f) *M*. *circinelloides* (*n* = 97, 92, 124, 78) in the presence of 50 nM LTR and imaged for 6 h. Data show the mean phagosomal LTR localisation time (min) ± SEM taken from three independent experiments. *n* = pooled number of phagosomes analysed across three independent experiments per fungal organism. At least 50 macrophages were analysed per mouse per fungal organism. ns = not significant, * *p* < 0.05, ** *p* < 0.01, *** *p* < 0.001, **** *p* < 0.0001 (one-way ANOVA).

Following PAMP-PRR interactions, fungal cells are internalised into a phagosome that undergoes sequential maturation. To measure the dynamics of the latter process, we assayed phagosome localisation to either LTR or LAMP-1. First, localisation to the commercially available dye LysoTracker Red-DND99 (abbreviated LTR, a marker of early phagosome maturation) was monitored for each individual phagosome. Quantitative analysis showed that phagosome maturation was significantly slower in knockout BMDM compared with WT BMDM (Fig 5). In most fungal co-cultures, early phagosome maturation was most strongly delayed in dectin-2^-/-^ BMDM, and this was particularly evident in *C*. *auris* co-cultures (Fig 5b). In this setting, there was a four-fold difference between WT and dectin-2^-/-^ BMDM in the time taken to attain phagosomal LTR positivity (mean ± SEM = 10.8 ± 0.7 min and 42.3 ± 5.1 min for WT and dectin-2^-/-^ BMDM, respectively).

Compared with WT BMDM, deficiency in FcRγ also correlated with delayed early phagosome maturation in most fungal-cultures. In contrast, deficiency in mincle only significantly affected LTR colocalisation in *C*. *albicans*, *C*. *auris*, and *S*. *cerevisiae* co-cultures, but had no effect in *Malassezia* (Fig 5d and 5e) and *Mucor* (Fig 5f) co-cultures. Together, these data suggest that for most species deficiency in FcRγ, dectin-2, and for selected species, mincle, led to delayed early phagosome maturation.

**Fig 6:**
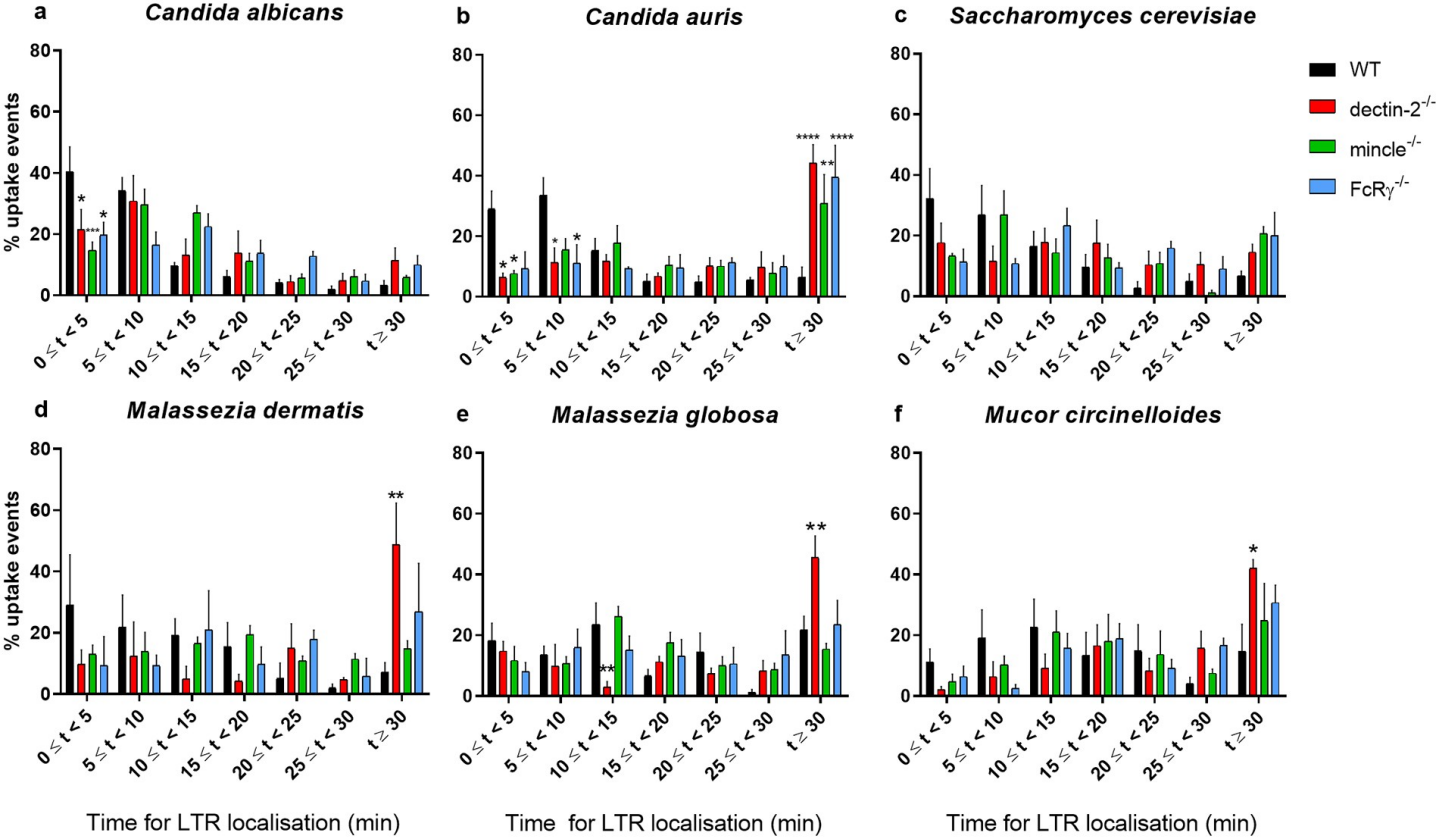
Deficiency in FcRγ or its associated receptors dectin-2 and mincle correlates with a greater percentage of phagosomes requiring longer time to become LTR positive. BMDM from WT, dectin-2^-/-^, mincle^-/-^, or FcRγ^-/-^ mice were inoculated with (a) *C*. *albicans* (*n* = 264, 267, 349, 298), (b) *C*. *auris* (*n* = 350, 366, 977, 287), (c) *S*. *cerevisiae* (*n* = 276, 201, 284, 231), (d) *M*. *dermatis* (*n* = 618, 760, 991, 264), (e) *M*. *globosa* (*n* = 545, 804, 820, 409), or (f) *M*. *circinelloides* (*n* = 97, 92, 124, 78) in the presence of 50 nM LTR and imaged for 6 h. Data show the mean percentage ± SEM of phagosomes requiring different lengths of time to localise with LTR, and are from three independent experiments. *n* = pooled number of phagosomes analysed across three independent experiments per fungal organism. At least 50 macrophages were analysed per mouse per fungal organism. * *p* < 0.05, ** *p* < 0.01, *** *p* < 0.001, **** *p* < 0.0001 (two-way ANOVA).

Early phagosome maturation was analysed in more detail by breaking down the first 30 min post-engulfment into shorter time segments and analysing the percentage of LTR-positive phagosomes for each segment. A trend was observed where the greatest percentage of phagosomes in WT BMDM took less time to localise with LTR compared with knockout BMDM (Fig 6). In most settings, this was significant within 10 min post-engulfment (Fig 6a and 6b). In contrast, only a minority of phagosomes in knockout BMDM localised with LTR at this early time post-engulfment. Consequently, in most fungal co-cultures, the greatest percentage of knockout phagosomes (in particular for dectin-2^-/-^ BMDM) took 30 min or longer to attain LTR positivity (Fig 6b, 6d, 6e and 6f). Therefore, these analyses suggest that while phagosomes in different BMDM were heterogeneous in their rate of maturation, deficiency in FcRγ, mincle, and dectin-2 in particular resulted in significantly delayed phagosome maturation in the majority of assessed phagosomes.

**Fig 7:**
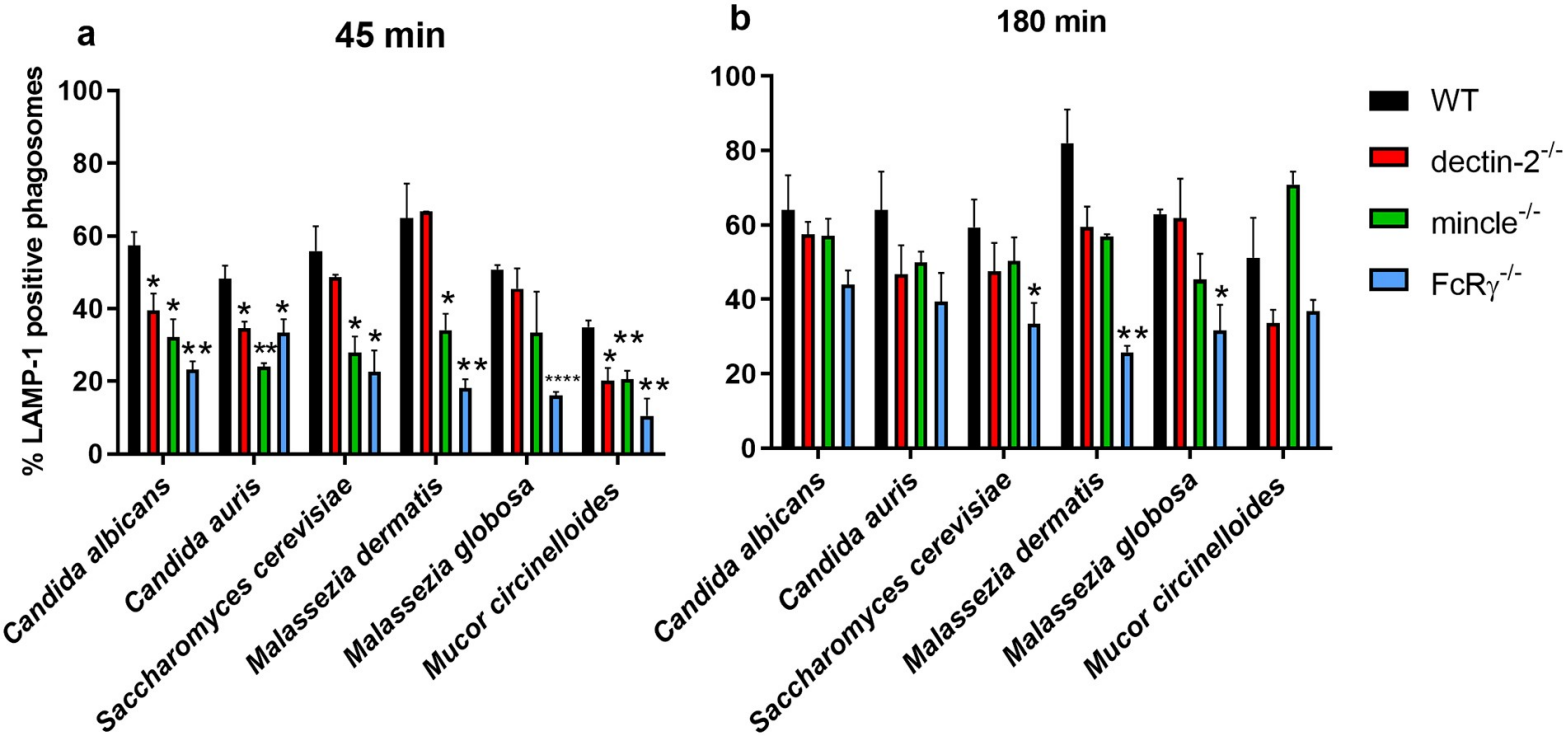
Deficiency in FcRγ or its associated receptors dectin-2 and mincle correlates with impaired phagosomal LAMP-1 positivity. BMDM from WT, dectin-2^-/-^, mincle^-/-^, or FcRγ^-/-^ mice were inoculated with various fungi for (a) 45 min or (b) 180 min and analysed for phagosomal LAMP-1 positivity. Data show the mean percentage ± SEM of LAMP-1 phagosome positivity from three independent experiments. At least 50 macrophages were analysed per mouse per fungal organism. * *p* < 0.05, ** *p* < 0.01, *** *p* < 0.001, **** *p* < 0.0001 (Student’s *t*-test).

Secondly, BMDM were inoculated with different organisms as described previously and stained with anti-LAMP-1. LAMP-1 is one of the two most abundant lysosomal membrane proteins (LAMP-1 and LAMP-2), and is a classical marker of the latter stages of phagosome maturation. At 45 min of co-culture, deficiency in dectin-2, mincle, and FcRγ correlated with a reduced percentage of LAMP-1-positive phagosomes compared with WT BMDM, and the magnitude of the difference depended on the organism studied (Fig 7a). These differences largely disappeared by 180 min of co-culture (Fig 7b). However, phagosomal LAMP-1 positivity was still significantly reduced in FcRγ^-/-^ BMDM compared with WT BMDM in co-cultures with *S*. *cerevisiae* and *Malassezia* spp. Together, these data suggest that deficiency in FcRγ, dectin-2, or mincle significantly impaired early phagosome maturation, which translated into impaired late phagosome maturation as observed for LAMP-1 phagosome positivity.

### Deficiency in FcRγ impacts macrophage viability in the presence of *M*. *circinelloides*

Phagosome maturation serves the purpose to inhibit further replication/dissemination of pathogenic cells. This is typically associated with fungal killing. However, in many cases, pathogens elaborate various virulence mechanisms to subvert killing/containment. Amongst other means, this could be through non-lytic escape [32], lateral transfer to neighbouring phagocytes [33], or physical or pyroptotic rupture of phagocyte membranes leading to lytic escape [18, 34]. In previous studies on *C*. *albicans*, death was measured by observing hyphal-mediated lysis of macrophages [2, 30, 35, 36]. In this study, however, the commercially available viability dye (YOYO-3) was used as a cell death marker. YOYO-3 is cell-impermeable, and only fluoresces when bound to exposed nucleic acids upon loss of membrane integrity.

**Fig 8:**
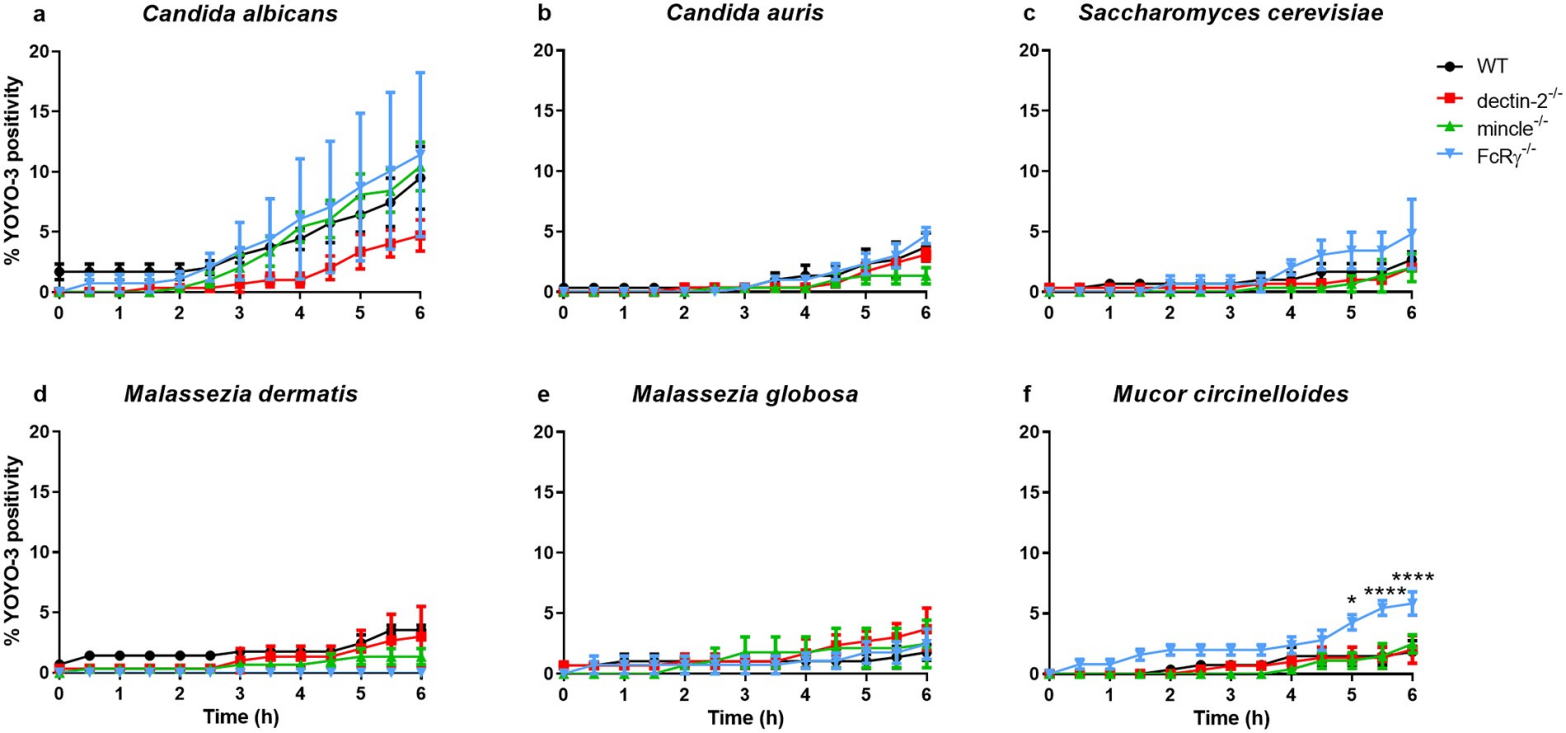
Deficiency in FcRγ or its associated receptors dectin-2 and mincle does not impact macrophage viability significantly. BMDM from WT, dectin-2^-/-^, mincle^-/-^, or FcRγ^-/-^ mice were inoculated with various fungi in the presence of 1 μM YOYO-3 and imaged for 6 h. Data show the mean percentage ± SEM of YOYO-3 positivity from three independent experiments. At least 50 macrophages were analysed per mouse per fungal organism. * *p* < 0.05, ** *p* < 0.01, *** *p* < 0.001, **** *p* < 0.0001 (two-way ANOVA).

By 6 h of imaging, deficiency in FcRγ, dectin-2, or mincle had little effect on macrophage viability, although it was more evident in *C*. *albicans* co-cultures (Fig 8a). The only exception to these observations was the case for *M*. *circinelloides* co-cultures, where %YOYO-3 positivity was significantly higher for FcRγ^-/-^ BMDM (Fig 8f). This difference started appearing after 5 h of imaging. Together, these data suggest that deficiency in FcRγ may be exploited by *M*. *circinelloides* to kill macrophages.

## Discussion

Signalling through PRRs constitutes a critical aspect of innate immunity. Whereas some PRRs play an exclusively signalling role (*e*.*g*. TLRs), others induce phagocytosis (*e*.*g*. MR), and still others do both (*e*.*g*. FcγRs) [37]. In this report, we used live cell video microscopy to assess the role of FcRγ in shaping the spatio-temporal dynamics of fungal phagocytosis. Using macrophages from mice deficient in the FcRγ chain itself, as well as dectin-2 and mincle (two CLRs that critically require FcRγ for optimal expression and function), we showed that these receptor knockouts were impaired in the phagocytosis of a range of organisms across the fungal kingdom. In particular, we report that loss of overall FcRγ expression, as well as that of dectin-2 or mincle, was associated with significantly reduced macrophage migration, engulfment, and phagosome maturation, but macrophage viability was largely unaffected in these knockouts. Our results are in agreement with previous studies, which have established the important role of these receptors in phagocytosis of both fungal and non-fungal cargo [20, 38–42].

Activation of and signalling via CLRs is particularly important for mounting successful immune responses to fungal infections. Apart from dendritic cell immunoreceptor (DCIR), CLRs of the dectin-2 cluster lack cytosolic hemITAMs for signal transduction, and therefore couple to adaptor proteins containing signalling motifs, such as DAP10, DAP12, or FcRγ [43] to mediate signal transduction. Dectin-2 in particular has been shown to be important in defense against disseminated *C*. *glabrata* [20], as well as *C*. *albicans* infections [38]. Mincle has been shown to be crucial in anti-*Candida* responses, as mice with a deficiency in mincle show significantly increased susceptibility to systemic candidiasis [22]. Further, mincle has been shown to be important for the recognition of, binding to, and defense against *Malassezia* spp. *In vitro* and *in vivo* analyses of cytokine/chemokine production by mincle^-/-^ BMDM revealed a significant impairment [44]. These studies thus highlight the key roles such CLRs play in mounting successful responses to fungal infections.

Our previous studies [2, 17–19, 30, 35, 45] have shown that factors such as fungal cell viability, morphogenesis, hyphal length, cell wall glycosylation status, and spatial orientation impact on the dynamics of phagocytosis. We also recently reported a role for dectin-2 in defense against systemic infection with *C*. *glabrata* [20]. Although deficiency in dectin-2 did not significantly impair migration or killing, we observed a significant impairment of uptake and phagocytosis. Because dectin-2 is an FcRγ-coupled CLR, these findings prompted us to hypothesise that FcRγ-coupling may be pivotal for shaping the dynamics of fungal phagocytosis.

We chose six organisms representing diverse mycological taxa to dissect the spatio-temporal dynamics of PRR-mediated phagocytosis using live cell video microscopy. We showed that deficiency in FcRγ or its associated receptors, dectin-2 and mincle, impaired macrophage migration. This impairment was strongest for dectin-2^-/-^ and FcRγ^-/-^ BMDM compared with WT BMDM, whereas mincle^-/-^ BMDM were more moderately impaired. In our previous study [20], we did not demonstrate a significant effect of dectin-2 deficiency on macrophage migration. Differences in the type of macrophage (*i*.*e*. peritoneal *vs* bone marrow-derived), activation status (*i*.*e*. prior priming or not with IFNγ), growth medium, and pathogen (*i*.*e*. *C*. *glabrata*) may account for such differences.

In parallel, we showed that knockout BMDM also exhibited reduced macrophage displacement compared with wild-type counterparts in most fungal co-cultures. This implies that FcRγ, dectin-2, or mincle deficiency directly impact the speed of macrophage migration. We did not closely examine this parameter in our previous studies [2, 18, 20, 35, 36, 45], and this is informative in that it describes both macrophage speed and extent of movement from original position in targeting various cargo. Furthermore, it is plausible that receptor deficiency in this case alters macrophage responses to chemotactic gradients, thereby resulting in reduced migration and displacement.

We reported previously that J774.1 macrophage-like cells incubated with glycosylation mutants of *C*. *albicans* showed improved migration but delayed engulfment [2]. In the present study, however, engulfment dynamics tracked migration dynamics. In most fungal co-cultures, we observed that all knockouts were significantly impaired in the rate of engulfment compared with WT BMDM. The magnitude of this response varied, and this is not surprising given the morphological diversity of the fungi under examination. *C*. *auris* yeast cells, for example, undergo rapid aggregation. *M*. *dermatis* and *M*. *globosa* are also clumpy by nature, and we routinely observed large numbers phagocytosed by BMDM even at the beginning of imaging. Therefore, some of the observed defects in engulfment may relate to such organism-specific differences. However, our data show that the reduced engulfment rate associated with deficiency in FcRγ, dectin-2, or mincle was replicable for multiple phylogenetically diverse fungi.

We demonstrate that deficiency in FcRγ, dectin-2, or mincle impaired phagosome maturation. The time from completion of engulfment until phagosomal localisation with LTR, a marker of acidified cellular compartments, was increased in the case of knockout BMDM. Phagosome maturation was particularly delayed in dectin-2^-/-^ BMDM, and this was clearest in co-cultures with *Malassezia* spp. and *M*. *circinelloides*. Upon examining the first 30 min post-engulfment in more detail, we observed that most phagosomes in wild-type BMDM were LTR-positive within 10 min of engulfment, which contrasted with phagosomes in knockout BMDM. Consequently, phagosomes in FcRγ-, dectin-2-, or mincle-deficient BMDM attained LTR positivity only at or after 30 min post-engulfment in most fungal co-cultures. These observations were reinforced by fixed cell staining assays, where delayed phagosomal LAMP-1 positivity in knockout BMDM compared with WT BMDM was observed.

Deficiency in FcRγ, dectin-2, or mincle did not greatly impact macrophage viability, with the exception of *M*. *circinelloides* co-cultures. Although not significant, it was noted that the magnitude of YOYO-3 positivity increased the most in *C*. *albicans* co-cultures. This partly mirrors previous observations wherein we investigated macrophage death via hyphal-mediated lysis using established methodologies [18, 30, 35, 36]. In our microscopic analyses, we observed that *C*. *albicans* rapidly formed hyphae. Therefore, it is arguable that hypha-mediated lysis contributed to much of the YOYO-3 positivity we observed.

Our *in vitro* experiments show repeatedly that loss of FcRγ, dectin-2, or mincle from murine BMDM compromises individual components of the phagocytic pathway, namely: migration (evidenced by mean track velocity and mean displacement); engulfment; and phagosome maturation (evidenced by phagosomal localisation to both LTR and LAMP-1).

Still, further work is required to identify the specific mechanisms underlying the observed outcomes. In particular, it would be critical to dissect which pathways FcRγ, dectin-2, and mincle specifically regulate to facilitate phagocytosis of fungal pathogens. Furthermore, future work should address how different fungal pathogens may interfere with these receptors’ function to survive being in the hostile phagosome and/or escape. *C*. *albicans*, for instance, is known to activate stress response pathways, including the Hog1, Cap1, and Hsf1 pathways, to counteract the hostile, microbicidal lumen of the phagosome [14, 15]. In addition, *C*. *albicans* activates the ATO gene family to regulate ammonia extrusion, thereby neutralising the phagosome and promoting hyphal growth [16, 46]. Moreover, we have previously shown that *C*. *albicans* interferes with Rab14 dynamics, another contributing factor to phagosome maturation delay.

Other studies have demonstrated an important role for the serine/threonine phosphatase calcineurin in mediating *M*. *circinelloides* pathogenicity [47]. Experiments comparing wild-type strains and counterparts deficient in the *cnaB* subunit of calcineurin showed that *M*. *circinelloides* spores, but not yeast-locked mutants, block or delay phagosome maturation. Interestingly, the authors reported that this was not entirely dependent on phagosomal pH modulation as had been observed with *C*. *albicans* [16, 46]. Therefore, it would be intriguing to examine how these and other mechanisms of phagosome maturation blockage/delay are specifically regulated by FcRγ, dectin-2, or mincle.

We observed a strong effect for dectin-2 deficiency on most parameters assayed in this study, which at times paralleled or was even greater than that of FcRγ deficiency. In an earlier study [21], stimulation of dectin-2^-/-^ and FcRγ^-/-^ bone marrow-derived dendritic cells (BMDC) with mannans had no observable differences on production of IL-12p40, IL-6, TNF, IL-1β, or IL-10. Here, dectin-2 was identified as an α-mannan receptor required for Th17 cell differentiation during systemic *C*. *albicans* infection, and further showed the importance of IL-17A, but not IL-17F, in mediating the protective response.

In conclusion, live cell video microscopy demonstrated how CLR-associated FcRγ-coupling influences the dynamics of fungal phagocytosis. We showed that deficiency in the CLRs dectin-2 and mincle, as well as their common adaptor FcRγ, led to multiple effects on phagocytosis, including reduced macrophage migration, displacement, engulfment time, and phagosome maturation, but largely no effects on macrophage viability.

## Supporting information

S1 Fig Tracking diagram of WT, dectin-2^-/-^, mincle^-/-^, and FcRγ^-/-^ BMDM in co-cultures with *C*. *albicans*. (TIF)

S2 Fig Tracking diagram of WT, dectin-2^-/-^, mincle^-/-^, and FcRγ^-/-^ BMDM in co-cultures with *C*. *auris*. (TIF)

S3 Fig Tracking diagram of WT, dectin-2^-/-^, mincle^-/-^, and FcRγ^-/-^ BMDM in co-cultures with *S*. *cerevisiae*. (TIF)

S4 Fig Tracking diagram of WT, dectin-2^-/-^, mincle^-/-^, and FcRγ^-/-^ BMDM in co-cultures with *M*. *dermatis*. (TIF)

S5 Fig Tracking diagram of WT, dectin-2^-/-^, mincle^-/-^, and FcRγ^-/-^ BMDM in co-cultures with *M*. *globosa*. (TIF)

S6 Fig Tracking diagram of WT, dectin-2^-/-^, mincle^-/-^, and FcRγ^-/-^ BMDM in co-cultures with *M*. *circinelloides*. (TIF)

S1 Vid BMDM phagocytosing *C*. *albicans* CAI4-CIp10. (AVI)
